# 3M-052 combined inhibitory anti-TNFR2 synergistically suppresses colon cancer progression

**DOI:** 10.3389/fimmu.2026.1749062

**Published:** 2026-06-03

**Authors:** Qianyu Jing, Junqian Luo, Quan Wan, Qianjun Jing, Xiefei Hu, Cui Nei, Xuelian Zhang, Yan Zhou, Jing Tang, Yongchao Wang, Yan Ding, Yujie Nie, Wei Li, Yingjie Nie

**Affiliations:** 1 Department of Clinical Laboratory, Chongqing Emergency Medical Center, School of Medicine, Chongqing University Central Hospital, Chongqing University, Chongqing, China; 2 The First People’s Hospital of Jinzhong, Jinzhong, China; 3 The University of Hong Kong-Shenzhen Hospital, Shenzhen, Guangdong, China; 4 Chongqing Medical University, Chongqing, China; 5 Guizhou Provincial People’s Hospital, Guiyang, China

**Keywords:** 3M-052, anti-TNFR2, colon cancer, immunotherapy, TCM

## Abstract

**Introduction:**

Immunotherapy is considered the most promising approach for achieving complete tumor clearance in cancer treatment. However, monotherapy has demonstrated limited clinical efficacy, leading to a growing consensus that combination immunotherapy—activating the immune system through multiple pathways—appears to be the optimal strategy for enhancing therapeutic outcomes. In this study, we investigated the efficacy and underlying mechanisms of the combination immunotherapy MT—comprising the TLR7/8 agonist 3M-052 and the inhibitory anti-TNFR2 antibody—in colon cancer (CRC).

**Methods:**

A CRC mouse model was established and divided into four treatment groups: the control group, the 3M-052 group, the anti-TNFR2 group, and the MT group. Tumour growth was monitored every three days. Flow cytometry was used to analyse the levels of CD8+ T cells and regulatory T cells (Tregs) in tumour tissue, as well as the differentiation of central memory T cells (Tcm) in the tumor-draining lymph nodes (TdLNs).

**Results:**

The results revealed that the combination therapy MT promoted CD8+ T cell infiltration and reduced Tregs level within the tumor, effectively suppressed colon cancer growth in mice, and induced long-term syngeneic tumor control by enhancing the differentiation of Tcm in TdLNs. Compared with monotherapy, combination therapy MT demonstrated a superior capacity to induce the differentiation of central memory CD8+ and CD4+ T cells in TdLNs, an effect that appears to be primarily mediated by 3M-052.

**Discussion:**

This study demonstrates that the combination of 3M-052 and the anti-TNFR2 antibody exerts a synergistic effect, effectively inhibiting CRC progression and inducing long-term immune memory. Overall, these findings support the view that MT combination therapy represents a potentially effective strategy.

## Introduction

Growing evidence suggests that the tumor immune microenvironment (TME) plays a crucial role in cancer immune escape and treatment resistance. Currently, immunotherapy that targets the TME and thus restores the anti-tumor effects of T cells is a hotspot in anti-tumor technology research (1). However, targeting one specific microenvironment in the TME alone may induce a series of changes in other specific microenvironments and related pathways (2). Combination therapies that simultaneously target multiple specific microenvironments may greatly benefit cancer treatment (1).

Regulatory T cells (Tregs), a key mediator in the TME, are abundantly present in many types of cancer tissues. Tumor necrosis factor receptor type II (TNFR2) is one of two receptors that mediate the biological functions of TNF (3–5), which is highly expressed on a variety of cancer cells, exhausted CD8+ T cells, and Tregs (6, 7), and is a critical molecule that participates in the activation, proliferation, and immunosuppressive function of Tregs (8, 9). Inhibitory antibodies targeting TNFR2 (anti-TNFR2) can effectively inhibit the proliferation of Tregs and promote their apoptosis, and have achieved excellent efficacy in a variety of mouse tumor models, representing a potential strategy for tumor therapy (3, 5, 10–12). Our preliminary research has demonstrated that anti-TNFR2 can inhibit TNF’s positive effects on Tregs and reduce the proportion of tumor-infiltrating Tregs in CRC (13).

Toll-like receptors (TLRs) are a family of pattern-recognition receptors, characterized by extracellular recognition domains that specifically recognize pathogen-associated molecular patterns, thereby initiating downstream signaling cascades that subsequently activate adaptive and innate immune cells, and play a crucial role in enhancing the antigen presentation capacity of antigen-presenting cells (APC) (14). Dendritic cells (DCs) are the most functional APCs and play a key role in cancer immunotherapy (15). However, a variety of molecules in the tumor microenvironment, such as IL-10, vascular endothelial growth factor, prostaglandin E2, etc., can inhibit the activation of DCs, thus unable to effectively activate T cells (15). TLR agonists enhance the expression of co-stimulatory molecules and antigen presentation on DCs, promote the shift of CD4+ T cell responses from Th2 to Th1, inhibit the Treg response, and ultimately enhance CD8+ T cell responses, and show potent anti-tumor activity in a variety of mouse tumor models (16–18). However, TLR agonists have limited application in cancer therapy. TLR9 agonists are poorly expressed on human monocytes and are therefore ineffective in clinical therapy (13). R848, a TLR7/8 agonist, is rapidly distributed to the circulation after intratumoral injection, producing systemic exposure and side effects (19). Therefore, the TLR7/8 agonist MEDI9197 (3M-052) has been created. 3M-052 has a lipid tail structure that is insoluble in water and does not readily spread throughout the body after intratumoral administration, and produces an immune response superior to that induced by R848 (20, 21).

In our previous studies, TLR agonists combined with anti-TNFR2 have demonstrated potential as an effective combination immunotherapy. Combination immunotherapy with HMGN1, R848, and anti-TNFR2 has significantly suppressed colon cancer growth in mice by activating dendritic cells (DCs) and inhibiting regulatory T cells (Tregs) (22). Treated mice have remained tumor-free and have developed long-term immune memory against the same tumor (22). To overcome the limitations of TLR agonists in clinical applications, we investigate the efficacy of 3M-052 combined with anti-TNFR2 therapy for colorectal cancer and explore the potential mechanisms by which this combination therapy induces long-term tumor control.

## Materials and methods

### Mice and culture of cell lines

The cell lines (CT26 and 4T1) were purchased from Pricella (Wuhan, China). CT26 and 4T1 cells were cultured in RPMI-1640 medium supplemented with 2 mM glutamine, 10% FBS, streptomycin (100 μg/ml), and penicillin (100 U/ml) at 37 °C under 5% CO_2_ conditions.

Female wild-type Balb/c mice aged 6 to 8 weeks were provided by Beijing Spefer Biotechnology Co., Ltd. (SCXK (Beijing) 2019-0008). Mice were kept in the SPF laboratory of the Experimental Animal Center at Guizhou University of Traditional Chinese Medicine (SYXK 2021-0005) and acclimated for 7 days before the experiment. The animal experiment (Approval No. EC Review (Animal): 2022-053) was approved by the Ethics Committee of Guizhou Provincial People’s Hospital, China.

### Extraction, culture, and functional analysis of myeloid-derived dendritic cells

Bone marrow cells extracted from the femur and tibia of mice were cultured for six days in RPMI-1640 complete medium with the addition of GM-CSF (20 ng/ml), IL-4 (10 ng/ml), 25 mM HEPES and 50 μM 2-mercaptoethanol, and were differentiated into mDCs. These mDCs (5×10^5^/ml/well) were treated with lipopolysaccharide (LPS), 3M-052, vehicle (anhydrous ethanol), anti-TNFR2 (20 μg/ml) for 24 hours, followed by flow cytometry to detect the expression levels of mature markers on the cell surface, qPCR to detect the mRNA expression levels of IL-12 and IL-6, and ELISA to detect the levels of IL-12, IL-6, IL-10, and IFN-γ in the supernatant. Next, the mDCs from each group were collected using a cell scraper for co-culture with splenic mononuclear cells (SPMCs).

### Extraction of splenic mononuclear cells

Mouse spleens were placed in culture dishes containing an appropriate volume of 1640 medium and ground. The resulting suspension was filtered through a 70 μm cell strainer to obtain a single-cell suspension. All cell suspensions were transferred to a 15 ml centrifuge tube that contained Ficoll solution, and a clear liquid interface was maintained. The tube was centrifuged at 800g for 20 minutes at 22°C with acceleration and deceleration both set to 1. The cloud-like cell layer at the interface of the liquid surface was aspirated using a pipette. The cells were washed with 10 ml PBS, centrifuged at 300g for 5 minutes, the supernatant was discarded, and the cells were resuspended in 1640 medium.

### Co-culture

The dendritic cells (1×10^5^/ml/well) after intervention and spleen mononuclear cells (1×10^6^/ml/well) were co-cultured for 72 hours in RPMI-1640 complete medium supplemented with 0.1 mM nonessential amino acids, 50 μM 2-mercaptoethanol, and 0.03% glutamine. Then, all cells were collected and co-cultured with cancer cells, the supernatant was collected, and the levels of IFN-γ and IL-2 were detected by ELISA.

The CT26 cell concentration was adjusted to 1×10^7^/ml. 5 μM CFSE dye was added and incubated for 15 minutes at 37 °C, 5% CO_2_. Cells were washed with PBS before proceeding to subsequent experiments. The SPMCs (1×10^6^/ml/well) after intervention and CT26 cells (2×10^5^/ml/well) were co-cultured for 48 hours in RPMI-1640 complete medium. Then, all cells were collected and apoptosis of CT26 cells was detected by flow cytometry.

### Cell apoptosis

All cells were collected, incubated with CD45 antibody for 15 minutes, washed with PBS, and then incubated with PI-FITC antibody for 15 minutes. Cells were collected using a flow cytometer.

### RT-qPCR

Total RNA was extracted using an RNA extraction kit, cDNA was generated using a reverse transcription kit, and RNA expression levels were assessed by real-time quantitative polymerase chain reaction (RT-qPCR) on the CFX Connect™ Optics Module (Bio-Rad). The primer sequences used in this study are shown in **Supplementary Table 2**.

### ELISA

ELISA experiments were performed using cell culture supernatants according to the reagent manufacturer’s instructions. Absorbance was measured using Epoch (Bio Tek Instruments). Data were analyzed using ELISACalc software.

### Construction of animal models and combination therapy

CT26 or 4T1 tumor cells (2 × 10^5^ cells/0.1 ml PBS) were injected subcutaneously into the right abdominal region of mice in the form of a single-cell suspension. When tumor volume reached 100 mm^3^, mice were treated according to the following treatment regimen: anti-TNFR2 (200 μg/200 μl PBS) was administered intraperitoneally on days 2 and 7. 3M-052 (20 μg/100 μl PBS) was administered intratumorally on days 1, 5 and 9. As a control group, the same dose of PBS was administered into mice using the same injection method. In the re-challenge experiment, the same number of CT26 cells were re-inoculated into the abdomen (left or right side) of tumor-free mice after treatment, and the same number of 4T1 cells were inoculated into the other side. In this study, “survival time” referred to the time required for the tumor to grow to 2000 mm³, which was the humane end point that triggered euthanasia. Subsequently, mice were euthanized via cervical dislocation. Tumor volume was monitored every 3 days using the following formula: Tumor volume = (length × width²)/2.

### Mouse serum collection

Whole blood samples from the orbital of mice were collected into tubes without anticoagulants. After the blood samples had completely coagulated, the tubes were centrifuged at 1,500g for 10 minutes. Then, the upper layer of serum was separated using a pipette into a new test tube and stored at −80 °C.

### Function testing of liver and kidney

ALT, AST, UREA, and CREA levels in mouse serum were detected using the Roche cobas^®^ 8000 modular analyzer series (Basel, Switzerland).

### Hematoxylin and eosin staining

The excised liver and kidneys were immersed in tissue preservation solution and sent to the Seville Laboratory (Wuhan, China) for tissue fixation, dehydration, paraffin embedding, sectioning, and staining. A microscope was used to record the morphological structure of the tissue.

### Flow cytometry analysis

Lymph nodes and spleens were ground and filtered through a cell strainer to obtain a single-cell suspension. Tumor tissues were cut into 1 mm³ fragments and digested for 3–4 hours in 40 mL of PBS containing 20 mg collagenase I and IV, 100 units hyaluronidase, and 4 mg DNase. The mixture was then filtered into a single-cell suspension by a cell filter. Cells were incubated with an antibody blocking Fc receptors for 15 minutes, then washed with PBS and incubated with appropriately diluted antibodies for 15 minutes. To determine the maturity of mDCs, cells were stained with CD11c, MHC II, CD80, and CD86 antibodies. To determine the percentage of CD8+ T cells and CD4+ T cells in the total T cell population, as well as the percentage of CD62L and CD44 on CD8+ T cells and CD4+ T cells, cells were stained with CD45, CD3, CD8, CD4, CD62L, and CD44 antibodies. Cells were incubated with a cell lysis buffer for 20 minutes. To determine the percentage of FOXP3+ CD4+ in the total T cell population, cells were stained with FOXP3 antibody. All cells were resuspended in 0.5 ml staining buffer prior to flow cytometry analysis. Data were collected using BD FACSCelesta™ (Becton, Dickinson and Company) and analyzed using FlowJo 10 software.

### Statistical analysis

All experiments in this paper were repeated three times. Statistical differences between groups were analyzed using one-way analysis of variance (ANOVA) and Log-rank tests in GraphPad Prism 7.0 software. All data are expressed as mean ± standard deviation (SD). A p-value < 0.05 was considered statistically significant.

## Results

### 3M-052 effectively promoted mDCs maturation *in vitro*

**Figure 1:**
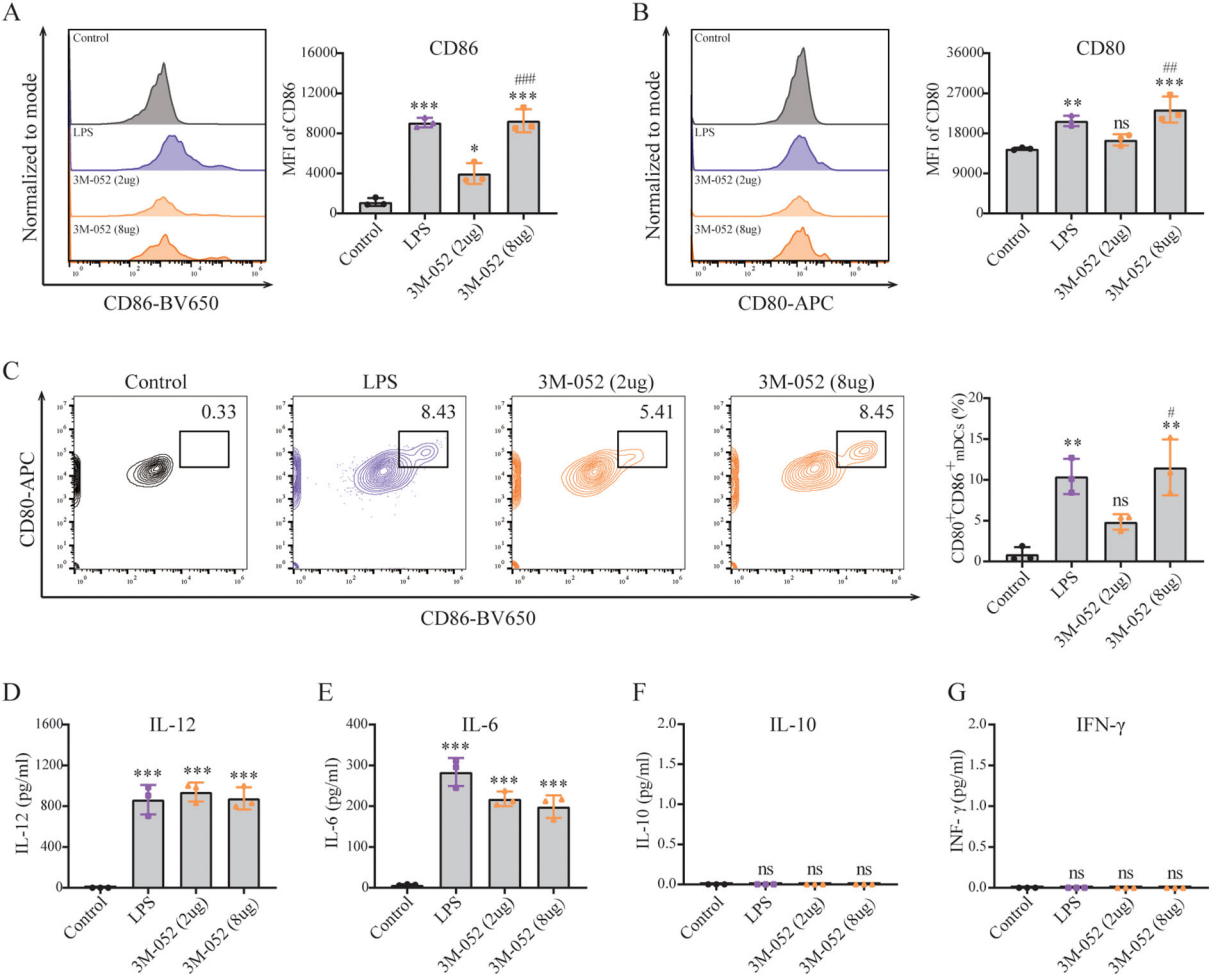
Effect of 3M-052 on mDCs. After 24 hours of drug intervention in mDCs, **(A)** Flow cytometric representation and MFI of CD86+ on mDCs; **(B)** Flow cytometric representation and MFI of CD80+ on mDCs; **(C)** Flow cytometric representation and proportion of CD86+CD80+ mDCs; **(D–G)** IL-12, IL-6, IL-10 and IFN-γ content in cell culture supernatants. Data are expressed as $\bar{X}$ ± *S*; *p < 0.05 vs control; **p < 0.01 vs control; ***p < 0.001 vs control. ^#^*p* < 0.05 vs 3M-052 (2 μg); ^##^*p* < 0.01 vs 3M-052 (2 μg).

First, we validated the effect of 3M-052 on mDCs *in vitro*, using LPS as a positive control. Flow cytometry was employed to detect markers of mDCs maturation (CD80, CD86). The flow cytometry gating strategies were shown in **Supplementary Figure 1A**. Results showed that after treatment with different concentrations of 3M-052 (2, 8 μg/mL), the MFI of CD86 and CD80 on mDCs significantly increased (**Figures 1A, B**), and the proportion of CD80+CD86+ mDCs markedly rose (**Figure 1C**). Furthermore, 3M-052 induced a marked increase in the secretion of the inflammatory cytokines IL-12 and IL-6 by mDCs (**Figures 1D, E**), while showing no effect on IL-10 and IFN-γ (**Figures 1F, G**). These data showed that 3M-052 effectively promoted mDCs maturation and secretion of IL-12 and IL-6 *in vitro*. Since the proportion of mature mDCs stimulated by 3M-052 at a concentration of 8 μg/mL showed no significant difference compared with the LPS group, subsequent experiments were conducted using 3M-052 at a concentration of 8 μg/mL.

### 3M-052-treated mDCs effectively enhanced the cytotoxic activity of splenic mononuclear cells

**Figure 2:**
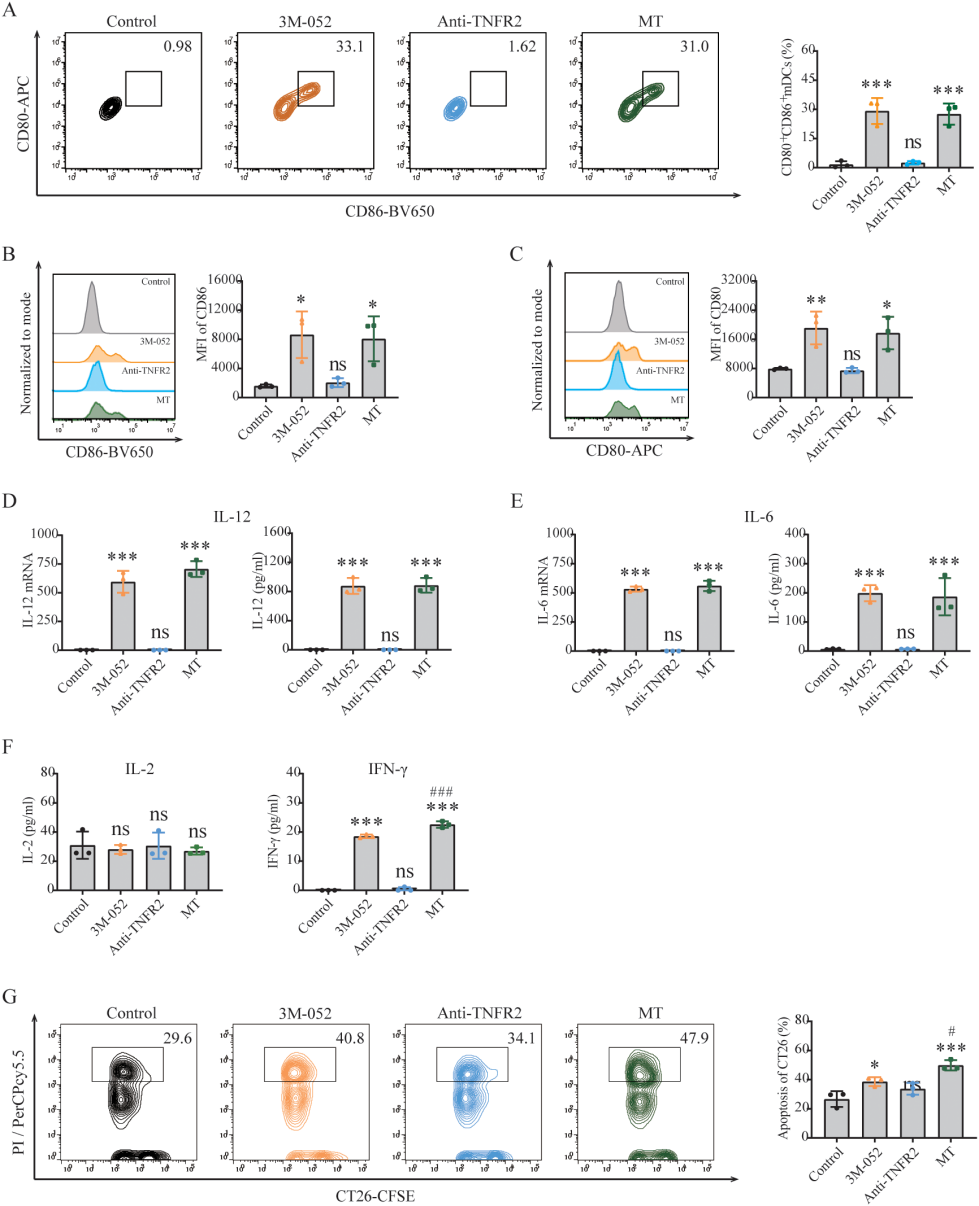
Effect of 3M-052 and anti-TNFR2 on mDCs. After 24 hours of drug intervention in mDCs, **(A)** Representative flow cytometric diagrams and proportions of CD86+CD80+ mDCs; **(B)** Flow cytometric representation and MFI of CD86+ on mDCs; **(C)** Flow cytometric representation and MFI of CD80+ on mDCs; **(D)** Relative mRNA expression levels and concentrations of IL-12 of mDCs; **(E)** Relative mRNA expression levels and concentrations of IL-6 of mDCs. Treated mDCs were co-cultured with mouse SPMCs for 3 days, **(F)** Concentrations of IL-2 and IFN-γ in cell supernatants. The cells after intervention were co-cultured with CT26 cells for 48 hours, **(G)** Flow cytometry diagrams and apoptosis rates of CT26 cells. Data are expressed as $\bar{X}$ ± *S*; *p < 0.05 vs control; **p < 0.01 vs control; ***p < 0.001 vs control; ^#^*p* < 0.05 vs 3M-052; ^###^*p* < 0.001 vs 3M-052.

Next, we explored whether anti-TNFR2 could enhance the effect of 3M-052 on mDCs *in vitro*. After treatment of mDCs with 3M-052 and/or anti-TNFR2 for 24 hours *in vitro*, there was no significant difference in the expression of CD80 and CD86 (**Figures 2A–C**), the relative mRNA expression and concentration of IL-12 and IL-6 (**Figures 2D, E**) between the anti-TNFR2 group and the control group. Compared with the control group, the expression of CD80 and CD86 (**Figures 2A–C**), and the relative mRNA expression and concentration of IL-12 and IL-6 (**Figures 2D, E**) were significantly increased in the 3M-052 and MT (3M-052+anti-TNFR2) groups, but there was no significant difference between the two groups. These data indicated that anti-TNFR2 had no effect on mDCs, but did not impair the effect of 3M-052 on mDCs. Next, we investigated whether the treated mDCs could activate the killing effect of SPMCs. After co-culturing the treated mDCs with SPMCs for 72 hours, the mDCs in the 3M-052 and MT groups significantly enhanced the ability of SPMCs to secrete IFN-γ, while IL-2 showed no significant change (**Figure 2F**). After further co-culture of the treated SPMCs with CT26 cells for 48 hours, apoptosis in CT26 cells was detected using flow cytometry. The flow cytometry gating strategies were shown in **Supplementary Figure 1B**. The result showed that the cytotoxic activity of SPMCs against tumor cells was significantly enhanced in both the 3M-052 group and MT group (**Figure 2G**). Compared with the 3M-052 group, the ability of SPMCs in the MT group to secrete IFN-γ and the cytotoxic activity against tumor cells were stronger, and the difference between the two groups was statistically significant (**Figures 2F, G**).

### MT significantly suppressed CRC growth and induced long-term tumor antigen-specific immunity in mice

**Figure 3:**
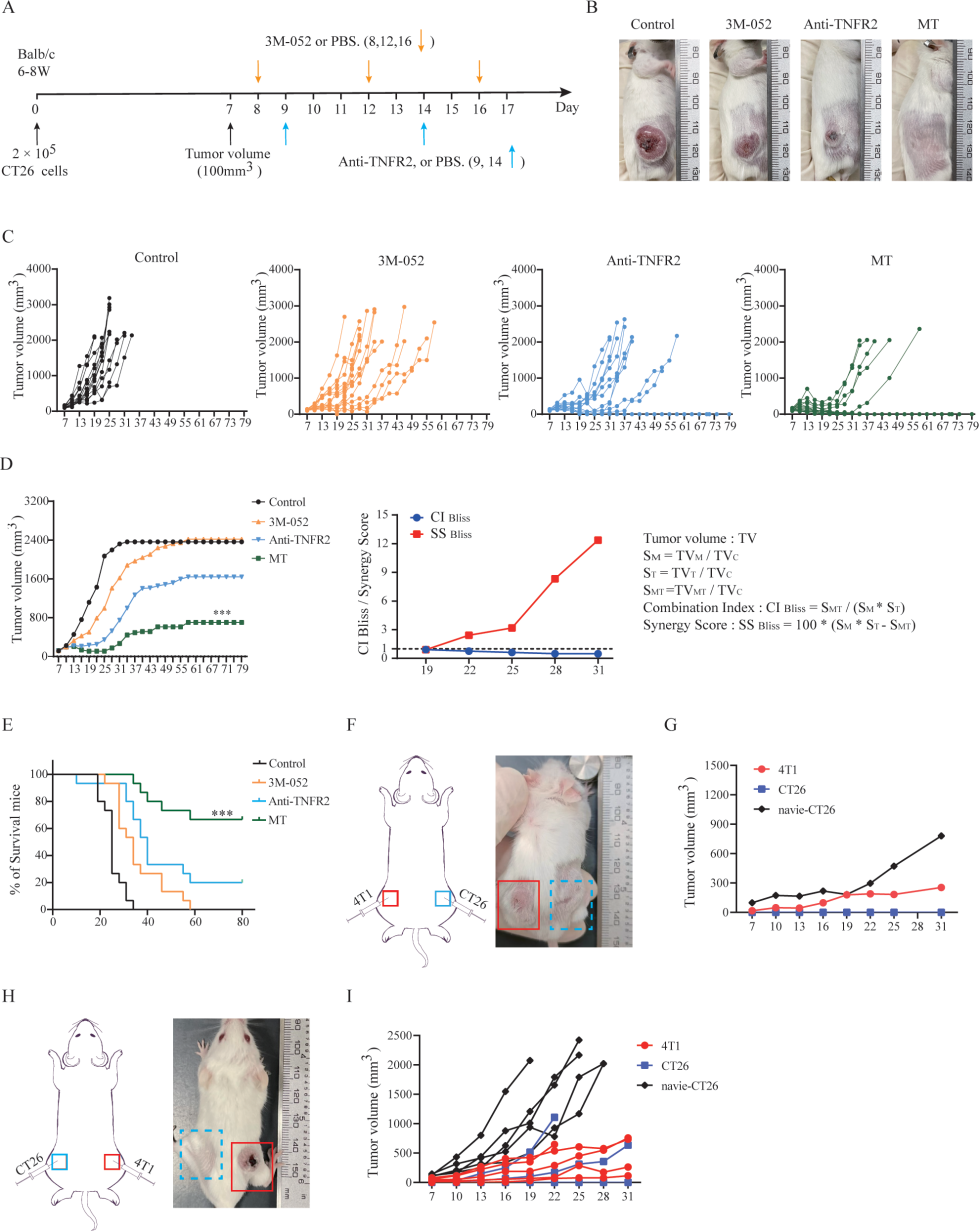
Therapeutic effect of combined therapy MT on CRC mice. **(A)** Schematic of treatment regimen: mice were treated with either 3M-052 (20 μg/100 μL) or anti-TNFR2 (200 μg/200 μL), the control group received an equivalent volume of PBS; **(B)** Representative schematic diagram of mice on day 19; **(C)** Tumor growth curves for mice (n=15); **(D)** Average tumor growth curves for mice (n=15), and the combination index (CI) and synergy score (SS) of MT therapy were evaluated using the Bliss independent model at different time points; **(E)** Representative survival curves for mice (n=15). Rechallenge experiment on the treated side of tumor-free mice, **(F)** The representative schematic diagram of mice on day 12; **(G)** Average tumor growth curves for mice (n=3). Re-challenge experiment on the opposite side of the treated side in tumor-free mice, **(H)** Representative schematic diagram of mice on day 12; **(I)** Tumor growth curves for mice (n=5). Data are expressed as $\bar{X}$; ***p < 0.001 vs control.

A mouse CRC model was established to evaluate the therapeutic efficacy of the combination therapy MT. Treatment plan was shown in **Figure 3A**. Results showed that compared with the control group and the single-drug group, the MT group significantly inhibited the tumor growth (**Figures 3B, C**). To evaluate the combined effect of 3M-052 and anti-TNFR2 in the treatment of CRC, we assessed the combination index (CI) and synergistic scores (SS) at different time points using the Bliss model. Results indicated that 3M-052 and anti-TNFR2 exhibited lower synergistic effects on day 19 (CI = 0.92, SS = 0.90), but demonstrated stronger synergistic effects starting from day 22 (CI = 0.76, SS = 2.41), which continued to intensify (**Figure 3D**). MT therapy effectively prolonged the survival period of tumor-bearing mice, maintaining an exceptionally high complete remission rate (66.7%) at the long-term observation point (day 79). This outcome significantly outperformed the best single-agent anti-TNFR2 therapy (20%), while no complete remissions were observed with single-agent 3M-052 therapy (**Figure 3E**). There were no significant differences in the weight of mice between groups (**Supplementary Figure 1C**). Next, to define whether these tumor-free mice developed long-term tumor antigen-specific immunity, they were subjected to re-challenge experiments with syngeneic tumors. As a control group, an equal number of CT26 cells were inoculated into the same part of normal mice. First, the same number of CT26 cells were re-injected into the treatment side of tumor-free mice, while the contralateral side received the same number of 4T1 cells (as control) (**Figure 3F**). Results showed that all mice developed 4T1 tumors but did not develop CT26 tumors (**Figure 3G**). Next, the inoculation sites for the two tumor cell lines were swapped: 4T1 cells were inoculated into the treatment side of tumor-free mice, while CT26 cells were inoculated into the contralateral side (**Figure 3H**). The results showed that all mice developed 4T1 tumors, whereas 60% of the mice did not develop CT26 tumors (**Figure 3I**). Our data showed that the combination of 3M-052 and anti-TNFR2 exhibited synergistic effects, effectively inhibiting the growth of colon cancer in mice and inducing long-term syngeneic tumor control.

### MT therapy induced CD8+ T cell tumor-infiltration and exhibited favorable cytotoxic characteristics

**Figure 4:**
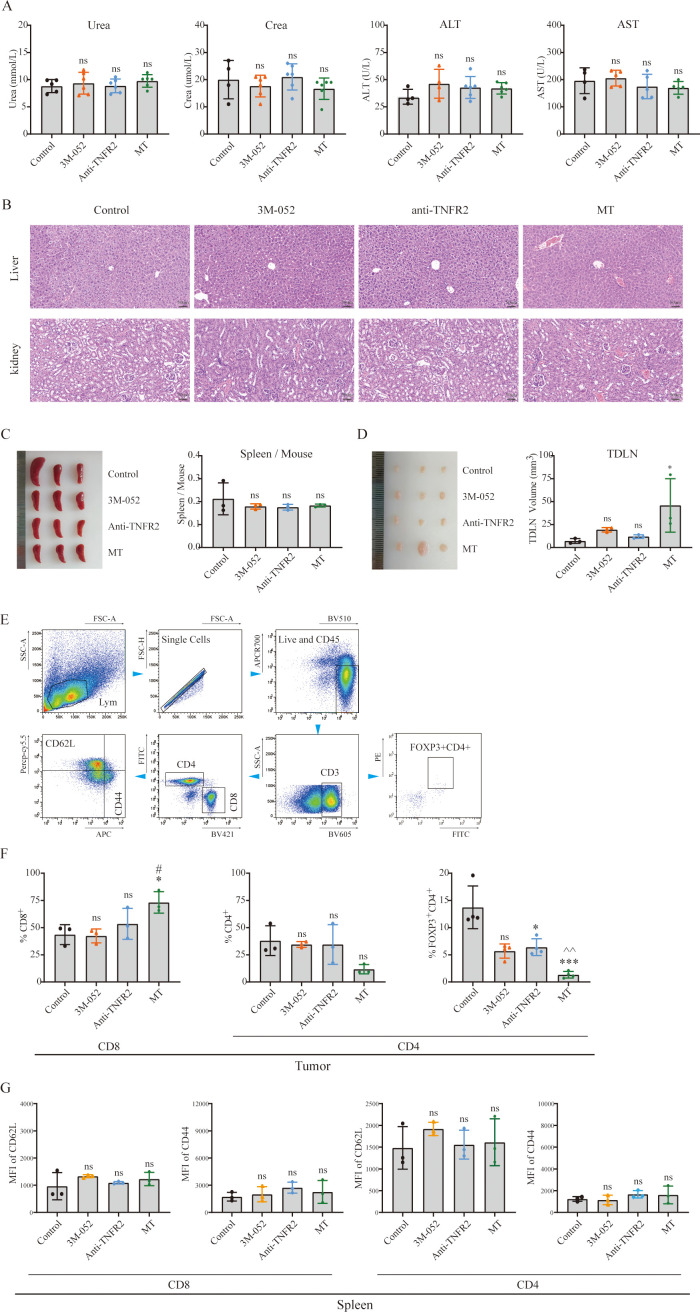
Toxicity evaluation of MT therapy and its effects on the immune system of mice. **(A)** Levels of Urea, Crea, ALT, and AST in serum of mice; **(B)** Representative HE-stained images of liver and kidney of mice; **(C)** Schematic diagram of the mouse spleen and the ratio of spleen to body weight in mice; **(D)** Schematic diagram and volume of the mouse TdLNs; **(E)** Flow cytometry gating logic; **(F)** Proportions of FOXP3+ CD4+ T cells, CD4+ T cells and CD8+ T cells within CD3+ T cells in tumors; **(G)** MFI of CD62L and CD44 in CD4+ T cells or CD8+ T cells in spleens. Data are expressed as $\bar{X}$±*S*; *p < 0.05 vs control; **p < 0.01 vs control; ***p < 0.001 vs control; ^#^*p* < 0.05 vs 3M-052; ^###^*p* < 0.001 vs 3M-052; ^^*p* < 0.01 vs anti-TNFR2.

To determine the toxicological characteristics of MT therapy on the body, on the day following the completion of the treatment regimen, the livers, kidneys, and peripheral blood of the mice were collected for analysis. Test results for liver and kidney function-related indicators showed that after MT treatment, no significant changes were observed in serum levels of ALT, AST, Urea, and Crea in the mice (**Figure 4A**). HE staining of the livers and kidneys revealed no significant structural abnormalities in either organ (**Figure 4B**). To further investigate the effects of MT therapy on the immune system, when tumor volume in the MT group significantly decreased compared with the control (on day 13), the spleen, lymph nodes, and tumor tissues were collected for analysis. In the MT group, the spleens showed no significant enlargement (**Figure 4C**), non-draining lymph nodes demonstrated no notable changes, and TdLNs exhibited significant enlargement (**Figure 4D**). The flow cytometry gating strategies were shown in **Figure 4E**. Compared with the control group, the proportion of CD8+ T cells was significantly increased, while the proportion of FOXP3+CD4+ T cells was significantly decreased in tumor tissue of the MT group (**Figure 4F**). In the spleen, the proportions of CD8+ T and CD4+ T cells showed no significant changes among different groups (**Supplementary Figure 1D**); the expression of CD62L and CD44 in both CD8+ T and CD4+ T cells also showed no significant alterations (**Figure 4G**).

### MT therapy effectively enhanced the differentiation of naïve and central memory T cells in TdLNs

**Figure 5:**
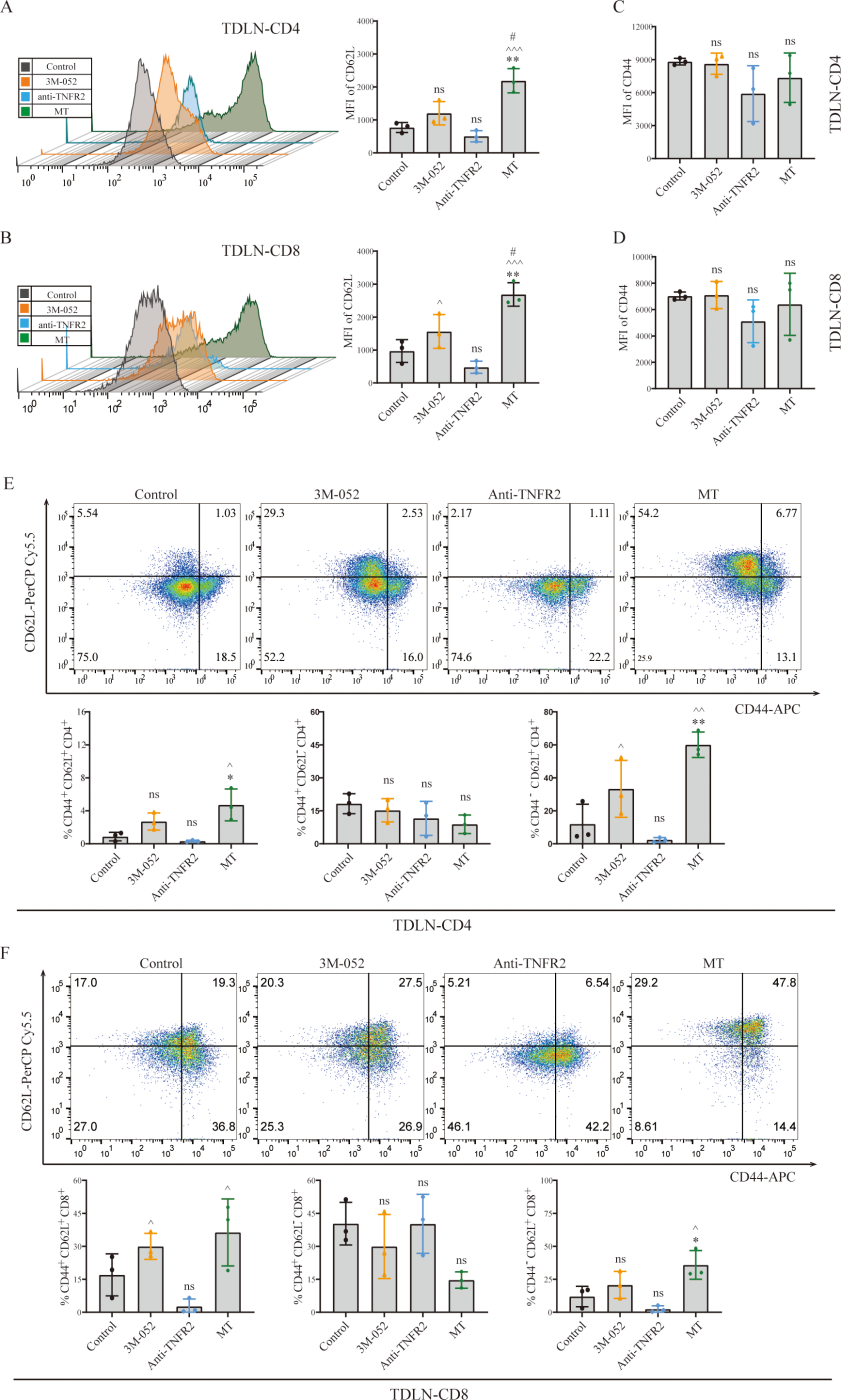
The effects of MT therapy on memory T cells in TdLNs. **(A)** Representative flow cytometric diagrams and MFI of CD62L+ in CD4+ T cells in TdLNs; **(B)** Representative flow cytometric diagrams and MFI of CD62L+ in CD8+ T cells in TdLNs; **(C)** MFI of CD44 in CD4+ T cells in TdLNs; **(D)** MFI of CD44 in CD8+ T cells in TdLNs; **(E)** Representative flow cytometric diagrams and proportion of CD62L-CD44+, CD62L+CD44-, and CD62L+CD44+ within CD4+ T cells in TdLNs; **(F)** Representative flow cytometric diagrams and proportion of CD62L-CD44+, CD62L+CD44-, and CD62L+CD44+ within CD8+ T cells in TdLNs. Data are expressed as $\bar{X}$±*S*; *p < 0.05 vs control; **p < 0.01 vs control; ***p < 0.001 vs control; ^#^*p* < 0.05 vs 3M-052; ^*p* < 0.05 vs anti-TNFR2; ^^*p* < 0.01 vs anti-TNFR2; ^^^*p* < 0.001 vs anti-TNFR2.

In TdLNs, the proportion of CD4+ T cells was significantly elevated in the MT group, while the proportion of CD8+ T cells was markedly reduced (**Supplementary Figure 1E**). In both CD8+ T and CD4+ T cells, CD62L expression was significantly elevated (**Figures 5A, B**), while CD44 expression remained unchanged (**Figures 5C, D**). Additionally, the percentages of naïve (CD62L+/CD44−) and central memory (CD62L+/CD44+) subsets were significantly increased in both CD8+ T and CD4+ T cells in the MT group (**Figures 5E, F**). Our experiments demonstrated that MT therapy effectively induced the differentiation of naïve and central memory among T cell subsets in TdLNs, and promoted CD8+ T cell infiltration into tumors.

## Discussion

DCs are a type of professional antigen presenting cell with the strongest antigen processing and presentation function, and they represent the central link in initiating and regulating the immune response (23). In mouse and human tumors, the expression of DC co-stimulatory molecules (such as CD80, CD86, etc.) correlates with CD8+ T cell differentiation (24). The high expression of co-stimulatory molecules and enhanced migratory capacity of mature DCs enable them to efficiently present antigens to T/B cells and initiate immune responses (23). However, DCs within the TME typically exist in an immature state, primarily responsible for antigen uptake and processing. Their low expression levels of co-stimulatory molecules on the cell surface lead to an inability to effectively activate immune responses (23). Consistent with other studies (21), we demonstrated that 3M-052 effectively promoted the maturation of DCs as well as the synthesis and secretion of the cytokines IL-6 and IL-12. Research indicates that in the immune response induced by DCs within lymph nodes, IL-6 promotes T cell activation, expansion, and survival (25, 26). IL-12 promotes Th1 cell differentiation and proliferation, activates and enhances CTL function, and facilitates memory cell formation, playing a crucial role in tumor immune regulation (27–29). Additionally, the anti-tumor immune response induced by DCs is dependent on CD8+ T cell activation and IFN-γ production (30, 31). IFN-γ plays a crucial role in the anti-tumor immune response by promoting the migration and activation of immune cells into the TME through transcriptional regulation of the chemokines CXCL9 and CXCL10 (32). We also demonstrated that DCs treated with the 3M-052 intervention effectively activated antitumor immune responses. Furthermore, anti-TNFR2 enhanced the antitumor immune responses induced by DCs treated with the 3M-052. This may be because the Tregs in splenic mononuclear cells had been eliminated by anti-TNFR2, thereby lifting the inhibitory effect of Tregs on CD4+ and CD8+ T cells and ultimately enhancing the antitumor cytotoxic activity of splenic mononuclear cells. Animal studies were consistent with *in vitro* experiments, demonstrating that MT therapy promoted CD8+ T cell infiltration into tumors, reduced the abundance of Tregs, and effectively inhibited the growth of colon cancer in mice.

TdLNs, as targets in tumor immunotherapy, play a crucial role in the antitumor immune response induced by immunotherapy and the formation of systemic memory phenotypes (33, 34). Central memory T cells (Tcm) are T cells that migrate to lymph nodes after antigen activation of naive T cells. They are characterized by persistence, memory function, and high proliferative capacity (35). Upon re-exposure to the same antigen, Tcm can be induced to rapidly generate large numbers of effective memory T cells (36, 37). In tumor immunotherapy, memory T cells are crucial for the persistence and effective tumor killing of T cells (38, 39). High-abundance tumor-infiltrating CD4+ Tcm cells are positively correlated with favorable prognosis in oral squamous cell carcinoma (40). Tumor-specific CD8+ T cells within TdLNs exhibit typical memory cell characteristics, such as expression of CD62L, CD127, and CD122, and are characterized as bona fide responders to anti-tumor immune responses mediated by immune checkpoint inhibitors (ICBs) (41). In this study, CD62L+ T cells and Tcm in TdLNs were significantly increased in the MT group. This may account for the antitumor effects and the long-term control of syngeneic tumors induced by MT treatment. Additionally, the significant increase in CD62L+ T cells and Tcm in TdLNs induced by MT combination therapy appears to be primarily mediated by 3M-052, as monotherapy with anti-TNFR2 did not affect CD62L+ T cells and Tcm in TdLNs. This may be attributable to the timing of TdLNs collection in this experiment. Overall, compared to monotherapy, combination therapy MT more readily induces the formation of systemic memory phenotypes in T cells within TdLNs.

## Conclusion

This study demonstrated through *in vivo* and *in vitro* experiments that the combination of 3M-052 with anti-TNFR2 produced synergistic effects, enhancing antitumor immune responses, effectively inhibiting colon cancer growth, and inducing long-term homologous tumor immune memory that may be attributed to the differentiation of memory T cells in tumor-draining lymph nodes (TdLNs). In summary, MT, as a highly promising cancer treatment modality, demonstrates potential antitumor effects against colon cancer.

## Data Availability

The original contributions presented in the study are included in the article/**Supplementary Material**. Further inquiries can be directed to the corresponding authors.
