# Supplementary figures and images for: 3M-052 combined inhibitory anti-TNFR2 synergistically suppresses colon cancer progression
Source: Front Immunol. 2026 Jun 3;17:1749062. doi: 10.3389/fimmu.2026.1749062 (PMC13272422; doi:10.3389/fimmu.2026.1749062)

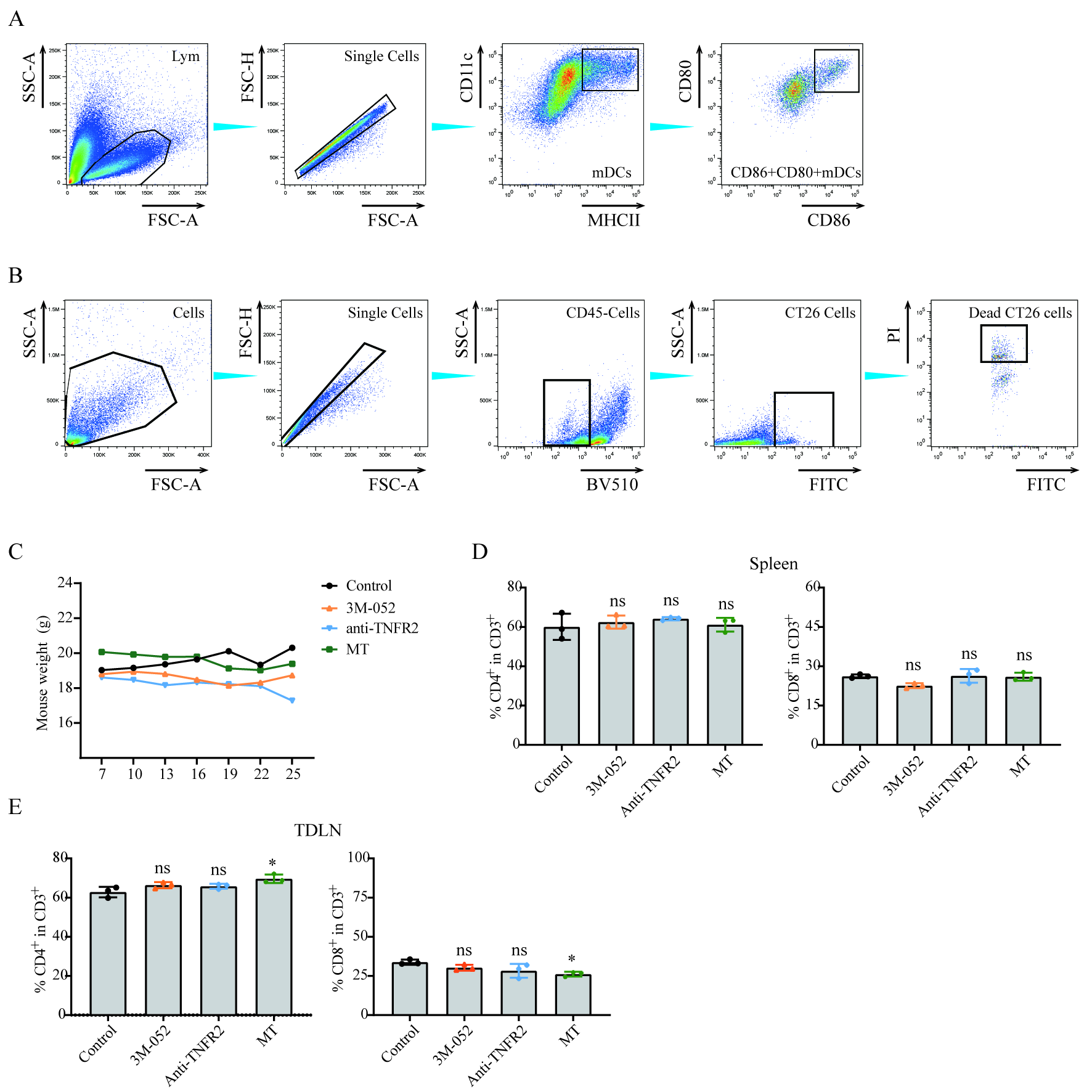

Supplement: Supplementary file 1 [file Image1.tif]
