# Supplementary material for: 3M-052 combined inhibitory anti-TNFR2 synergistically suppresses colon cancer progression
Source: Front Immunol. 2026 Jun 3;17:1749062. doi: 10.3389/fimmu.2026.1749062 (PMC13272422; doi:10.3389/fimmu.2026.1749062)
Supplement: Supplementary file 2 [file DataSheet1.docx]

**Supplementary Table S1.**

**Chemicals**

| Reagents used in this study |  |  |  |
| --- | --- | --- | --- |
| **Compounds/chemicals** | **Product code / clone number** | **Company** | **Place of production** |
| Telratolimod (3M-052) | HY-109104 | MedChenExpress | Monmouth Junction, NJ, USA |
| Anti-mouse TNFR2 (CD120b) antibody (clone TR75-54.7) | BE0247 | BioXCell | W. Lebanon, NH |
| Fetal bovine serum (FBS) | 164210 | Pricella | Wuhan, China |
| Penicillin-streptomycin stock solutions | C100C5 | New Cell & Molecular | Wuhan, China |
| Trypsin-EDTA (0.25%) | G4010 | Servicebio | Wuhan, China |
| RPMI-1640 medium with L-Glutamine | C11875500BT | GIBCO BRL | Grand Island, NY, USA |
| Nonessential amino acids 100× | 11140-050 | GIBCO BRL | Grand Island, NY, USA |
| Glutamine | 3108004 | GIBCO BRL | Grand Island, NY, USA |
| BV510-Rat anti-mouse CD45 | clone 30-F11 | BD Biosciences | Franklin Lake, New Jersey, USA |
| BV605-Rat anti-mouse CD3 | clone 17A2 | BD Biosciences | Franklin Lake, New Jersey, USA |
| BV421-Rat anti-mouse CD8 | clone 53-6.7 | BD Biosciences | Franklin Lake, New Jersey, USA |
| BV421-anti-mouse CD11c | Clone HL3 | BD Biosciences | Franklin Lake, New Jersey, USA |
| APC-anti-mouse CD80 | CloneS15049I | BD Biosciences | Franklin Lake, New Jersey, USA |
| BV650-anti-mouse CD86 | Clone GL1 | BD Biosciences | Franklin Lake, New Jersey, USA |
| FITC-anti-mouse CD4 | clone GK1.5 | BioLegend | California, USA |
| Anti-mouse CD16/32 | clone 93 | BioLegend | California, USA |
| Percp-cy5.5-anti-mouse-CD62L | MEL-14 | BioLegend | California, USA |
| PE-anti-mouse FOXP3 | CloneR16-715 | BD Biosciences | Franklin Lake, New Jersey, USA |
| APC-anti-mouse/human-CD44 | IM7 | BioLegend | California, USA |
| Percp-cy5.5-anti-mouse MHC II | M5/114.15.2 | BioLegend | California, USA |
| Zombie NTRTM Dye |  | BioLegend | California, USA |
| Annexin Ⅴ-FITC/PI Apoptosis Kit | AK10398 | Elabscience Biotechnology | Wuhan, China |
| RNA Rapid Extraction Kit | DP430 | ESScience | Shanghai, China |
| PrimeScrip RT reagent Kit with gDNA Eraser (Perfect Real Time) | RR047 | TaKaRa | Kyoto, Japan |
| SYBR Premix Ex Taq II (Tli RNaseH Plus) | RR820 | TaKaRa | Kyoto, Japan |
| Recombinant Murine GM-CSF | 315-03 | Thermo Fisher Scientific | Waltham, USA |
| Recombinant Murine IL-4 | 214-14 | Thermo Fisher Scientific | Waltham, USA |
| Mouse IL2 ELISA kit | EMC002QT96 | Neobioscience | Shenzhen, China |
| Mouse IFN-γ ELISA kit | EMC004.96 | Neobioscience | Shenzhen, China |
| Mouse IL6 ELISA kit | EMC005.96 | Neobioscience | Shenzhen, China |
| Mouse IL12p70 ELISA kit | EMC006.96 | Neobioscience | Shenzhen, China |
| Mouse IL10 ELISA kit | EMC005.96 | Neobioscience | Shenzhen, China |
| 2-Mercaptoethanol | M301573 | Aladdin | Shanghai, China |
| Human Peripheral Blood Lymphocyte Isolation Solution (Prepared by Ficoll) | P8900 | Solarbio | Beijing, China |
| HEPES | H8090 | Solarbio | Beijing, China |
| Collagenase I | C8140 | Solarbio | Beijing, China |
| Collagenase Ⅳ | C8160 | Solarbio | Beijing, China |
| DNase I | D8071 | Solarbio | Beijing, China |
| Hyaluronidase | H8030 | Solarbio | Beijing, China |
| DMSO | D8371 | Solarbio | Beijing, China |
| ALT Activity Assay Kit | 1.02.1203 | Fosun Pharma | Shanghai, China |
| AST Activity Assay Kit | 1.02.1003 | Fosun Pharma | Shanghai, China |
| Urea Assay Kit | 3030-717(S) | Ningbo Ruiyuan | Ningbo, China |
| Crea Assay Kit | 3020-717(S) | Ningbo Ruiyuan | Ningbo, China |

**Supplementary Table S2.**

| Primer | Sequence |
| --- | --- |
| Mouse IL12 | Forward：CTGTGCCTTGGTAGCATCTATG  Reverse：GCAGAGTCTCGCCATTATGATTC |
| Mouse IL10 | Forward：ACTCTTCACCTGCTCCACTG  Reverse：GCTATGCTGCCTGCTCTTAC |
| Mouse IL6 | Forward：AGTTGCCTTCTTGGGACTGA  Reverse：TCCACGATTTCCCAGAGAAC |
| Mouse actin | Forward：ACGCATGTACGTAGCCATCC  Reverse：CTCTCAGCTGTGGTGGTGAA |


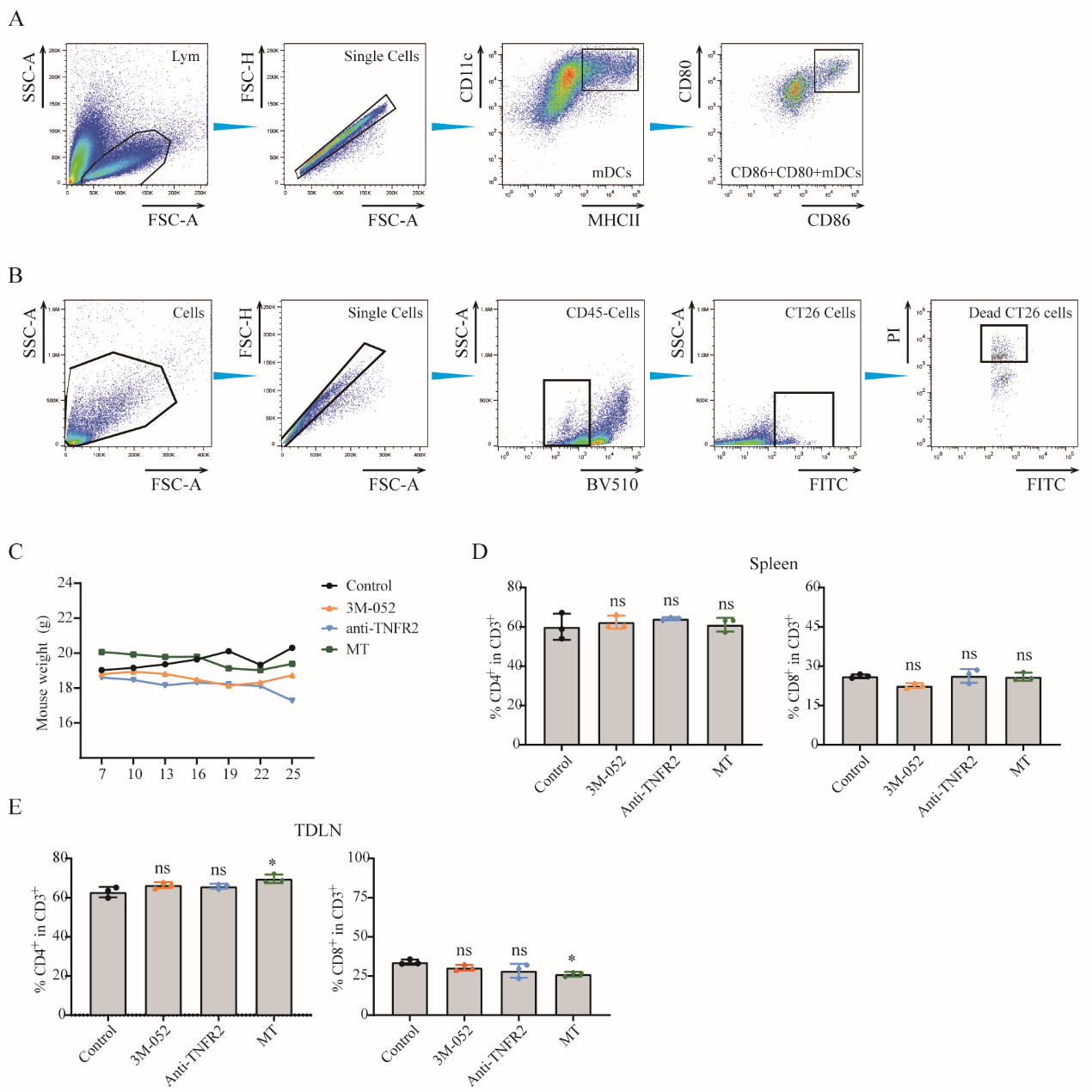


Supplementary Figure1

(A) Flow cytometry gating logics of mDCs; (B) Flow cytometry gating logics for co-culture of SPMCs and CT26 Cells; (C) Body weights of mice in each group during animal experiments; (D) Proportions of CD4+T and CD8+T within CD3+T cells in spleen; (D) Proportions of CD4+T and CD8+T within CD3+T cells in TDLN. Data are expressed as 𝑋̅±𝑆; * *p* < 0.05 vs control.
